# Determination of the source of SHG verniers in zebrafish skeletal muscle

**DOI:** 10.1038/srep18119

**Published:** 2015-12-11

**Authors:** William P. Dempsey, Nathan O. Hodas, Aaron Ponti, Periklis Pantazis

**Affiliations:** 1 Department of Biosystems Science and Engineering (D-BSSE), Eidgenössische Technische Hochschule (ETH) Zurich, 4058 Basel, Switzerland; 2 Pacific Northwest National Laboratory, Richland, Washington, USA

## Abstract

SHG microscopy is an emerging microscopic technique for medically relevant imaging because certain endogenous proteins, such as muscle myosin lattices within muscle cells, are sufficiently spatially ordered to generate detectable SHG without the use of any fluorescent dye. Given that SHG signal is sensitive to the structural state of muscle sarcomeres, SHG functional imaging can give insight into the integrity of muscle cells *in vivo*. Here, we report a thorough theoretical and experimental characterization of myosin-derived SHG intensity profiles within intact zebrafish skeletal muscle. We determined that “SHG vernier” patterns, regions of bifurcated SHG intensity, are illusory when sarcomeres are staggered with respect to one another. These optical artifacts arise due to the phase coherence of SHG signal generation and the Guoy phase shift of the laser at the focus. In contrast, two-photon excited fluorescence images obtained from fluorescently labeled sarcomeric components do not contain such illusory structures, regardless of the orientation of adjacent myofibers. Based on our results, we assert that complex optical artifacts such as SHG verniers should be taken into account when applying functional SHG imaging as a diagnostic readout for pathological muscle conditions.

Second harmonic generation (SHG) is a nonlinear optical process where two photons interact with a highly ordered, non-centrosymmetric material (that lacks a central point of symmetry) to generate a single photon with half the wavelength or double the frequency of the incident photons^1^,^2^. The efficiency of SHG production in a given material depends upon a material property known as the second-order nonlinear susceptibility tensor, χ^2^. SHG-derived contrast has a particularly attractive property relevant to biological imaging. The signal is tunable, meaning that specific illumination wavelengths can be chosen to minimize (i) background photons (e.g., from tissue autofluorescence) as well as (ii) spectral overlap with other contrast agents being imaged in parallel (e.g., fluorescent labels)^3^,^4^,^5^,^6^.

To produce detectable amounts of SHG signal, the SHG photons generated by different molecules must be in phase, leading to constructive interference of the signal. Certain components such as collagen, microtubules, and muscle myosin arrays possess sufficient order to fulfill these strict symmetry constraints. These biological structures generate detectable SHG signal, enabling them to be imaged in a label-free manner^7^,^8^. For instance, endogenous SHG contrast has been taken advantage of to reconstruct cell lineages and to study cell division patterns in early zebrafish embryos^9^.

The coherent nature of SHG has proven especially relevant when visualizing myosin bundles within the sarcomeres of striated muscle^8^. SHG signal has the capability to provide structural information about muscle fibers by highlighting individual sarcomeres within intact tissue^10^. Because structure and function are tightly linked for sarcomeres in striated muscle tissue, the ability to gain insight into the organization and structure of these biomolecule arrays has implications in clinically relevant imaging. For example, endogenous SHG contrast has been examined in medically relevant imaging, where underlying structural changes in SHG-capable biomolecule arrays can result from disease progression, such as in muscular dystrophy^11^,^12^ and cancer^13^.

One prominent SHG signal motif that is common in endogenous SHG imaging of skeletal muscle in both the zebrafish and the mouse are so-called “SHG verniers” — ‘Y’-shaped patterns and curved distortions in SHG signal between sarcomeric units in a myofiber (Fig. 1). These vernier pattern signals can be seen in both transverse and longitudinal optical sections. Muscle verniers were first described as staggered sarcomeres between adjacent myofibers in isolated *Rana temporaria* frog muscle fibers in the late 1950s^14^. More recently, researchers have observed comparable vernier patterns using SHG microscopy^12^,^15^,^16^,^17^, and some have postulated that SHG verniers represent the results of physical myofiber remodeling and tissue regeneration after muscle trauma^12^. Though the physiological origin of these signal patterns is uncertain, SHG verniers have been proposed to provide evidence for the progression of neuromuscular disease^12^. In this work, we present and validate a theoretical model that characterizes the SHG signal emanating from thick filaments of adjacent muscle cells, asserting that, in many cases, SHG verniers are optical illusions.

## Results

### Theoretical analyses suggest that SHG verniers are optical artifacts

**Figure 2:**
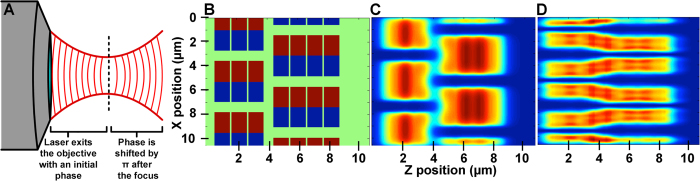
Theoretical calculations reveal dramatic differences between the signatures of SHG and TPEF signal. (**A**) This illustration depicts light being focused by an objective, where the sample plane is depicted using a dashed black line. Photons from the 2P laser exit the objective at a particular phase. At the focus, the phase shifts by π. (**B**) In a hypothetical piece of muscle tissue, two adjacent myofibers are modeled as organized arrays of rectangular “blocks” of myosin. The opposing orientation of the myosin filaments on either side of the M-line is represented as a color difference in the image, where the myosin block in one orientation is blue while the myosin across the M-line in the opposite orientation is in red. The green background represents the unlabeled and low contrast-producing exterior muscle environment. In this case, the laser is assumed to propagate horizontally across the hypothetical tissue. (**C**) Results of a simulation of TPEF emanating from fluorescently labeled myosin bundles within the sarcomere. The signal peaks toward the center of the thick filaments (deep red) and decreases outward (dark blue represents zero signal). There is little signal overlap between the staggered myofibers in this case, and the only gaps in signal are between the thick filaments themselves (the sarcomeric I bands). (**D**) Results from a simulation of endogenous SHG signal propagating from the aligned sarcomeric myosin. Unlike with TPEF, there is a clear enhancement in signal between the staggered myofibers, resulting from the Guoy phase shift of the Gaussian beam illumination at the focus. Thus, vernier patterns seen within the musculature of intact zebrafish embryos and larvae are likely a result of this optical artifact.

To investigate the source of SHG vernier patterns in muscle tissue, we constructed a simplified numerical simulation of an SHG microscopy experiment. We first applied our theoretical characterizations by modeling hypothetical muscle fibers *in silico*. In this model, we manipulated the geometry of adjacent synthetic muscle cells to determine the possible SHG signal patterns that may arise when illuminated with focused laser light (see Methods). To act as a standard for comparison of SHG signal from the muscle myosin, we also added a fluorescent component to the model, specifically considering the myosin thick filaments to be fluorescently labeled in addition to being SHG capable. The simplified hypothetical muscle tissue in our model is made up of two adjacent myofibers with multiple sarcomeric units (Fig. 2) organized at a length scale comparable to embryonic zebrafish skeletal muscle. In the model, we determined a single wavelength of illumination with which to compare SHG and two-photon-excited fluorescence (TPEF) from the myosin bands, settling on *λ* = 0.850 μm, which is the same wavelength that we subsequently used to generate SHG signal within zebrafish muscle tissue (see below). Additionally, we assumed an NA of 0.8 for the objective and 0.55 for the condenser in our simulations, as these are reasonably standard values when performing high resolution laser scanning imaging in many biological applications.

Our calculations yielded striking differences in TPEF (Fig. 2C) and SHG (Fig. 2D) signal. When adjacent myofiber sarcomeres were in a staggered orientation, TPEF signal is uniform within individual thick filaments, and maximal TPEF signal is visible at the M-line. The calculated SHG signature differs from the TPEF image in two important ways. First, SHG signal is absent at the M-line, which is a typical property of SHG signal arising from muscle fibers^8^, since the symmetry breaking conditions are eliminated at the M-line^18^. Consequently, two bright bands are present in the SHG signal for each sarcomere. Second, SHG is detected even from regions devoid of any myosin, diagonally between the myofibers, and this signature remains regularly ordered. These extra regions of SHG signal can be explained by considering the Gouy phase of the laser^19^, where the phase of light is shifted by π through the focus (Fig. 2A). Because we constructed the simulation to have the neighboring fibers to be out of phase by π, contributions of the Gouy phase result in constructive interference when the focus of the laser is directly between the two fibers. The results of the simulation suggest that SHG verniers and comparable distorted SHG signals may in fact be illusory, especially when compared to predicted TPEF signal from fluorescently labeled myosin, which mirrors the actual sarcomeric banded pattern more closely.

### Differing SHG and TPEF signal patterns within intact zebrafish muscle verify the illusory nature of SHG verniers

If verniers were in fact optical artifacts of the SHG process, we should be able to see differing signal in staggered muscle fibers when comparing SHG and TPEF images. Fluorescent emission from fluorescently labeled sarcomere components would show no increased fluorescence between fibers and would not have curvature or bifurcations in the signal, while SHG curvature and verniers would be prominent in the same imaging plane (Fig. S1), depending on the relative orientation of adjacent muscle cells (Fig. S2). To test this hypothesis, we performed imaging experiments on intact (i.e., not dissected or sectioned) zebrafish larvae. Zebrafish were chosen as specimens because of their small size and high degree of transparency during early development. Additionally, there are many established immunohistochemistry protocols and transgenic lines that make visualizing endogenous zebrafish structures particularly tractable. Further, the striated muscle compartments of embryonic and larval zebrafish are easy to distinguish, are simple in organization, and can be imaged completely intact using SHG and fluorescence microscopy (Fig. S3).

**Figure 3:**
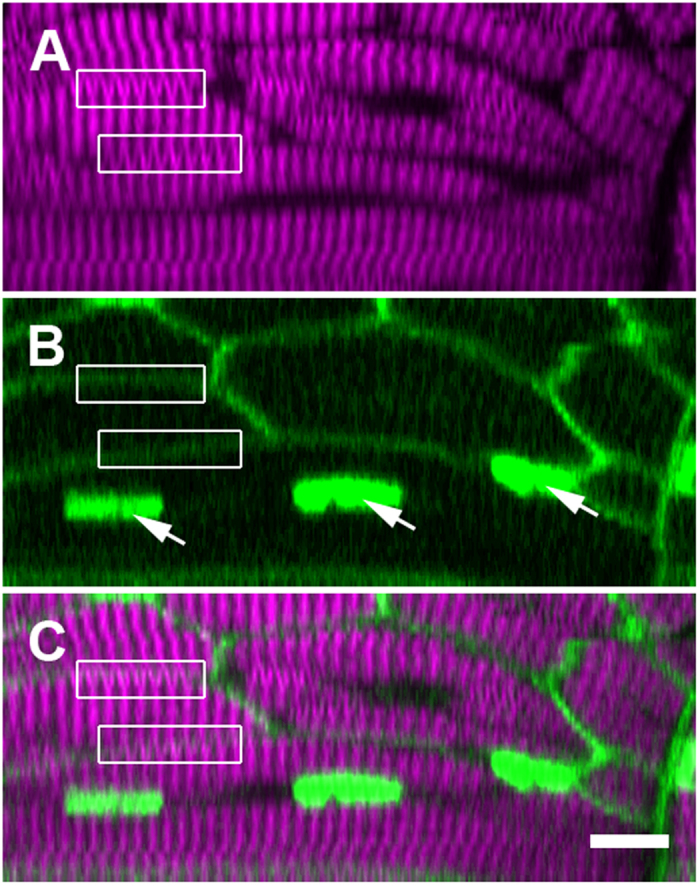
SHG verniers seem to cross intact membrane boundaries, indicating their illusory origin. These panels display a single ‘xz’ cross section within a trunk somite of a fixed 5 dpf zebrafish larva, where the membranes and nuclei are FP labeled (see Supporting Information, Methods). (**A**) Verniers (‘Y’-shaped) are visible within much of the thick filament-derived SHG signal (magenta). (**B**) Fluorescent reporter expression (green) illuminates the plasma membranes (diffuse borders) of the several visible muscle fibers as well as the nuclei within one particular cell (arrows). (**C**) The merged image of fluorescence and SHG signal shows that many SHG verniers seem to cross the physical membrane boundaries of the muscle cells (boxed regions, compare to the same boxed regions in panel **A**,**B**). Sarcomeric components should not connect across adjacent cell boundaries under normal physiological circumstances, especially in an unperturbed WT larva. Scale bar: 10 μm.

Since the model predicts that illusory SHG verniers have the potential to cross the plasma membrane boundaries of adjacent muscle cells (illustrated in Fig. S1B), we first attempted to capture this phenomenon by visualizing myosin SHG and membrane fluorescence in parallel within intact zebrafish embryos. Fluorescent cell labeling was facilitated by the use of PhOTO-N transgenic zebrafish larvae, where the membranes and nuclei of all cells within the embryo are ubiquitously labeled with spectrally separable fluorescent proteins^20^,^21^. We noticed SHG verniers that crossed intact membrane boundaries in 7-day post fertilization (dpf) zebrafish larvae (boxed regions in Fig. 3). Since sarcomere components are not connected across membrane boundaries, these data provide an initial confirmation of our theoretical model.

**Figure 4:**
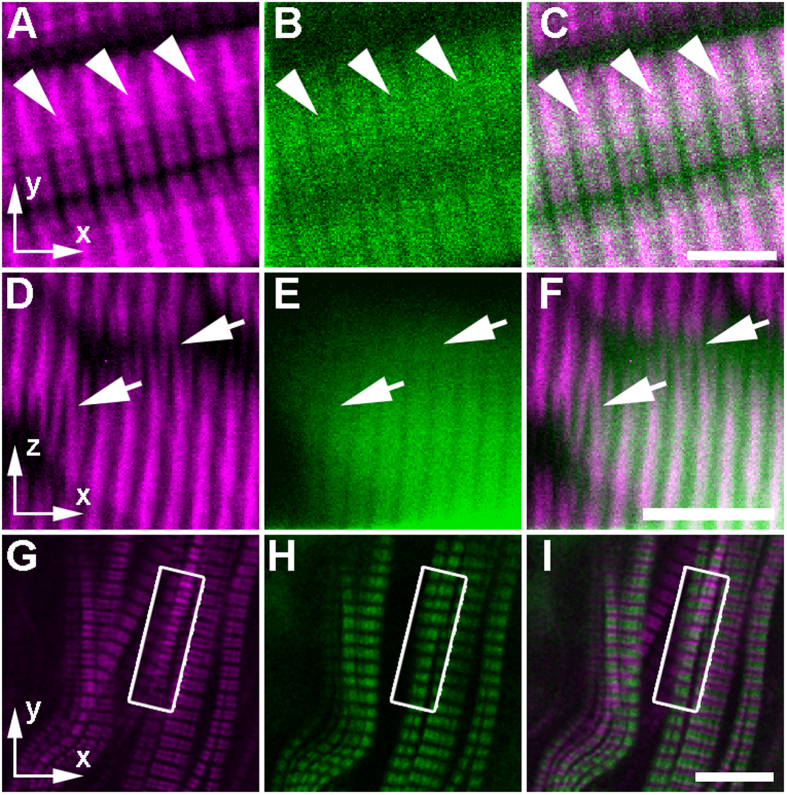
Apparent curvature in the sarcomere structure is only visible within the SHG channel when imaging fluorescently labeled sarcomeric proteins in zebrafish embryos. (**A**–**F**) In preparation for imaging, zebrafish larvae at 7 dpf were fixed and sparsely immunostained with anti-myosin MF20 primary and goat-anti-mouse-AF488 secondary antibodies (see Methods). In sagittal optical sections, (**A**) SHG signal (magenta) can be single banded and bifurcated, even within a single sarcomere (arrowheads), while (**B**) the TPEF signal (green) from each individual sarcomere can be seen as a single wide-band with gaps only at the I bands of the sarcomere (where myosin is absent), as predicted by the theoretical model. (**C**) The merged image shows the colocalization of SHG and TPEF signal within the thick filaments. In transverse optical sections, (**D**) adjacent myofibers seem to be linked by myosin verniers (arrows) in the SHG channel. (**E**) In the AF488 fluorescence channel (green), the banding pattern does not bifurcate at all (see the arrows pointing to the same regions as in (panel **D**). (**F**) In the merged image, the SHG signal and the fluorescent signal overlap within the labeled myofiber but do not show the same vernier signal pattern (arrows). Thus, the vernier patterns are only present in the SHG channel. (**G**–**I**) In this single sagittal optical section of the superficial slow muscle layer of a laterally mounted, fixed 5 dpf *Gt(tpm3-citrine)*^*ct5a*^ zebrafish larva, the thin filaments are visible, because endogenous tropomyosin 3 is fluorescently tagged. The box in each panel indicates the region where distortions appear in the SHG channel alone. (**G**) In the SHG channel, verniers can be seen as local curvature in the sarcomeric banding pattern. (**H**) In contrast, there is no curvature in the TPEF banding pattern, indicating that the sarcomeres are not actually physically distorted in this region. (**I**) This is demonstrated further in the merge of (panels **G**,**H**), where the distorted SHG vernier signal seems to overlap with the adjacent fluorescence band within the same muscle fiber. Scale bars: (**A**–**C**), 5 μm; (**D**–**I**), 10 μm.

Subsequently, we designed experiments to interrogate whether appreciable signal differences could be seen between SHG and fluorescence signal within intact WT zebrafish musculature. We fluorescently labeled sarcomeric components to ascertain whether verniers were present in the SHG image alone. Initially, myosin was fluorescently labeled in 7 dpf larvae that were fixed and immunostained (see Methods). In this case, the myosin-derived SHG co-localizes with the TPEF from the fluorophore-conjugated secondary antibody (Fig. 4A–F). In longitudinal optical sections, we noticed that — as predicted by the theory and from previous analyses of constructive interference at the M line from the literature^10^,^22^ — SHG from the sarcomeres can bifurcate even without the formation of verniers across muscle cell boundaries. This signal distortion occurs at the M line (Fig. 4A), whereas the TPEF signal remains even and only displays expected gaps in signal corresponding to the sarcomeric I bands, which are devoid of myosin (Fig. 4B). In transverse optical sections within mosaically immunostained samples, many SHG verniers seemed to link other adjacent myofibers (Fig. 4D). TPEF signal always ended at the boundary between muscle cells (Fig. 4E), while illusory SHG verniers crossed into adjacent cells (arrows in Fig. 4D–F).

In a second experimental condition, we wanted to ensure that the SHG verniers that we had seen were not an artifact of the myosin antibody labeling. In an effort to visualize labeled thin filaments within sarcomeres, we took advantage of the FlipTrap transgenic line *Gt(tpm3-citrine)*^*ct5a*^ established by Trinh *et al.*^23^, which is characterized by a yellow FP Citrine^24^ insertion into the endogenous locus of the gene that encodes the Tropomyosin alpha-3 chain (tpm3), a regulatory protein for actin-myosin contraction that is a component of the sarcomeric thin filaments within slow muscle tissue^25^. The TPEF signal from Citrine appears between the SHG signal bands in the sarcomeres (Fig. 4G–I). When the slow muscle was well aligned, there were few staggered fibers and few SHG verniers. However, when the muscle in a somite was slightly distorted as a consequence of zebrafish twitching that was frozen in time after fixation, we noticed a vernier pattern-like deflection in the SHG signal (Fig. 4G). As expected from the theoretical model, the fluorescence channel does not show these distortions (Fig. 4H). Taken together, the theoretical predictions are corroborated by experimental data, where fluorescence and SHG signal patterns of fluorescently labeled thick and thin filaments differ, typically when imaging adjacent myofibers with staggered sarcomeres.

### Reconstructing illusory SHG verniers synthetically using TPEF signal patterns as a guide

To account for the complex endogenous SHG signal patterns that arise in whole zebrafish tissue, we expanded our initial simplified SHG model into arbitrary geometries. We aimed to recapitulate vernier patterns in real tissue, validating the expected differences in TPEF versus SHG signal seen in the original theoretical analysis (Fig. 2). Our sample in this analysis was a fixed and immunostained 5 dpf zebrafish larva. An anti-myomesin antibody specifically labeled the M-line of each sarcomere in the tissue with appreciable contrast. We imaged the fixed larva and obtained a high-resolution optical volume containing both endogenous SHG from the muscle myosin as well as M-line specific TPEF from the antibody label. We segmented the TPEF signal from the immunostained zebrafish and generated a matrix of three-dimensional M-line positions within each sarcomere. Each M-line served as a three-dimensional starting point for the generation of a synthetic SHG pattern (see Supplementary Methods). Importantly, we maintained the length of each opposing thick filament emanating from the M-line within the normal physicological range of 0.6–1 μm^26^.

**Figure 5:**
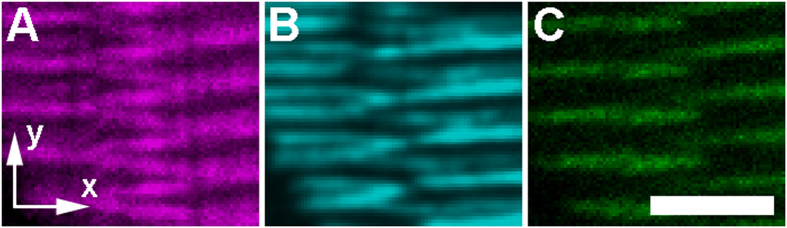
A theoretical model built upon fluorescence data agrees with actual vernier pattern SHG signal in whole mount zebrafish tissue. A fixed 5 day post fertilization zebrafish larva was immunostained with an anti-myomesin antibody, which selectively labels the M-line of skeletal muscle sarcomeres. A full 3D volume was imaged (see Supplementary Methods), and a region of interest with apparent SHG verniers was selected to test the theoretical model. (**A**) SHG signal (magenta) within a single optical section near the center of the imaged 3D volume shows clear vernier pattern SHG signal between two adjacent myofibers. (**B**) Synthetic SHG signal (cyan) was generated using our theoretical model (see Theory), starting with the (**C**) anti-myomesin TPEF signal (green) as a reference point for the M-line location and extent of each sarcomere. Scale bar: 5 μm.

Within this volume, vernier pattern signal in the endogenous SHG channel (Fig. 5A) crosses cell boundaries and links adjacent thick filaments. Synthetic SHG data (Fig. 5B) was calculated using only the 3D structure of each sarcomere as defined by the position of the TPEF signal (Fig. 5C) in the tissue, which did not cross into adjacent cells. The resulting synthetic data showed a striking qualitative resemblance to the true SHG signal, especially in the shape, number, and extent of connections seen between adjacent sarcomeres. Even though the 3D modeled sarcomeres did not physically connect adjacent cells, we still saw SHG verniers crossing over cell boundaries. Thus, using only the 3D structure of intact muscle tissue as a guide, our computational model successfully generated illusory SHG vernier signal spanning between adjacent staggered sarcomeres.

## Discussion

In an effort to uncover the source of SHG verniers within intact muscles, we implemented a theoretical model, paying special attention to the consequences of the phase coherence of SHG propagation and the Guoy phase shift of a laser at the sample focus. Unlike fluorescent signal within the sarcomere, which always retains a banded pattern that is evenly spaced and unconnected, SHG signal patterns can be distorted, depending on the orientation of the incoming laser as well as the organization of adjacent muscle fibers. Our analysis of phase coherence and cancellation proved to be invaluable for discovering this optical artifact. Comparable phase considerations have benefited other nonlinear microscopic approaches, such as coherent anti-Stokes Raman scattering microscopy^27^,^28^. We confirmed the illusory nature of SHG verniers described in the theoretical model by comparing SHG and fluorescence signal from fluorescently labeled sarcomeric components. Finally, using TPEF signal as a guide, we determined the 3D structure of sarcomeric repeats in a small volume of intact muscle tissue and verified that illusory SHG verniers can link adjacent cells in both synthetic and experimental data. Since only a small portion of a full somite was segmented for estimating the synthetic SHG signal, we only used the model for qualitative comparisons of SHG in the tissue.

Although our model predicts the occurrence of SHG verniers when adjacent sarcomeres are staggered, we do not rule out the possibility that some fraction of vernier patterns are a result of physical remodeling processes in the muscle cells, as has been previously shown in isolated rat and Xenopus muscle tissue^16^. Researchers should however be cautioned not to implicate all SHG vernier patterns as actual physical deformations in muscle tissue. Our model and our corroborating experimental evidence should guide the growing efforts to use endogenous SHG imaging as a diagnostic tool for the discovery and subsequent analysis of muscle structure and function-related disease progression (as in refs 11, 12 and 29): a strong focus on the underlying photophysical principles of SHG is necessary to distinguish between physical and illusory perturbations in compromised tissues. Ultimately, quantitative characterizations — of the particular orientation and extent within a tissue, for example — of even illusory structures like SHG verniers may represent a tractable approach for analyzing neuromuscular disease progression in humans, especially as new minimally invasive SHG microendoscopy protocols are formulated and refined (as in ref. 30).

## Methods

### Zebrafish husbandry

Zebrafish were raised, hatched, injected, and maintained in a house colony as previously described^31^. All animal procedures detailed were performed in accordance with official animal care guidelines and were approved by the Veterinary Department of the Canton of Basel-Stadt (Switzerland).

### Immunohistochemistry

Wild type (WT) or *Gt(tpm3-citrine)*^*ct5a*^ (23) transgenic zebrafish (*Danio rerio*) embryos were obtained by incrossing males and females from the same genetic background. These embryos were allowed to develop for ~18–20 hours before being treated with 1 × 1-phenyl 2-thiourea (PTU, Sigma Aldrich; St. Louis, MO) to inhibit pigment formation for the following few days of development. PTU medium was exchanged once every day or two. In the days before imaging, the embryos were kept in egg water^31^ in a Petri dish and kept in a 28 °C incubator.

To fix the zebrafish, the embryos/larvae were first anesthetized in 0.015–0.03% MS-222 (Finquel, Argent Laboratories; Redmond, WA) and then placed in 4% methanol-free formaldehyde (Thermo Scientific; Waltham, MA) on ice. After ∼5 min, embryos were allowed to continue to fix at room temperature on a nutator (BD Diagnostics; Sparks, MD) for 1 hour before being washed in Ca^2+^/Mg^2+^-free 1× PBS 3 times (15 min, each). *Gt(tpm3-citrine)*^*ct5a*^ zebrafish and the WT zebrafish were immediately prepared for imaging.

Embryos that were stained with antibody were first blocked overnight at 4 °C in 1× PBS (Ca^2+^/Mg^2+^-free) + 1% dimethyl sulfoxide (DMSO) + 1% bovine serum albumin (BSA) + 1.5% Triton X-100 detergent (referred to as PBSTB). Anti-sarcomere myosin (MF20, hybridoma supernatant) and anti-myomesin (mMaC Myomesin B4) primary antibodies were obtained from the Developmental Studies Hybridoma Bank at the University of Iowa. Primary antibodies were incubated on a nutator at room temperature in PBSTB with the whole-mount zebrafish for 2.25 hours (1:3 dilution). Embryos were washed 3 times (15 min each) in PBSTB before being suspended in PBSTB and treated with goat anti-mouse IgG secondary antibodies (1:200 dilution) conjugated to Alexa Fluor 488 nm (AF488, Invitrogen; Grand Island, NY). The embryos were incubated with the secondary antibody protected from light at room temperature on a nutator for 2.5 hours. After 3 washes (10 min each), the embryos were prepared for imaging.

### Zebrafish imaging

For all fixed zebrafish imaging experiments, zebrafish were embedded in 1% low melting point agarose in 30x Danieau’s solution^32^ and were imaged on an inverted geometry Zeiss (Jena, Germany) laser scanning microscope (LSM) 710 or 780 system with two-photon capabilities (Coherent, Chameleon Ti/Sapphire laser source). Images were obtained using an LD C- Apochromat 40 × 1.1 NA water immersion objective, an LD LCI Plan-Apochromat 25x/0.8 NA Imm Corr DIC multi-immersion objective, or a C-Apochromat 63x/1.2 NA oil immersion objective (Zeiss; Jena, Germany). Custom filters (kindly provided by Semrock, Inc.; Rochester, NY) placed in the transmitted light path on the microscope enabled detection of SHG signal (a 680/KP short-pass blocking filter preceding a 417/60 bandpass filter). Two-photon illumination wavelengths between 850 nm and 890 nm were used in these experiments, since they produced optimal SHG in zebrafish striated muscle.

Fluorescence signal was detected in the epi-direction using standard detection filter settings for each fluorophore. Successive optical sections were taken using the Zeiss LSM to cover large volumes of the muscle compartments and to locate vernier patterns from staggered muscle fibers deep within the tissue. We define “longitudinal” (x-y) optical sections as images that are taken parallel to the long axis of the zebrafish, while “transverse” (x-z) optical sections are images that are taken perpendicular to the long axis of the zebrafish.

Images were processed in Imaris (Bitplane, AG), ImageJ (NIH), and Adobe Photoshop CS3 (Adobe Systems), where minor linear brightness/contrast adjustments and filtering (small area median filter) were performed.

From the large 3D volume multichannel (i.e., SHG and TPEF signal) datasets, sub-volumes were extracted that contained both vernier-pattern SHG signal and appreciable TPEF signal. All of the subsequent algorithms for segmentation and estimation of the surface normal vectors were performed using a script written in MATLAB (MathWorks; Natick, MA, USA). Implementation of the full 3D theoretical model to recapitulate the experimental SHG data using the sarcomeric TPEF signal was performed using a custom script in the open source Julia language (julialang.org). Poisson distributed background noise was simulated in the model data using a custom MATLAB script.

Because SHG verniers are often found deep within the muscle compartments within zebrafish somites where light and antibody penetration is reduced, TPEF signal emanating from the immunostained sarcomeric components was low. The resulting low signal-to-noise ratio TPEF images were difficult to process using automatic segmentation algorithms, so we instead took advantage of a segmentation toolkit to perform manual segmentation of the data^33^. We averaged the hand-annotated segmentation results performed by the individual authors, and black-and-white masks of the manual analysis data were used for further processing.

## Additional Information

**How to cite this article**: Dempsey, W. P. *et al.* Determination of the source of SHG verniers in zebrafish skeletal muscle. *Sci. Rep.* **5**, 18119; doi: 10.1038/srep18119 (2015).

## Supplementary Material

Supplementary Information

## Figures and Tables

**Figure 1:**
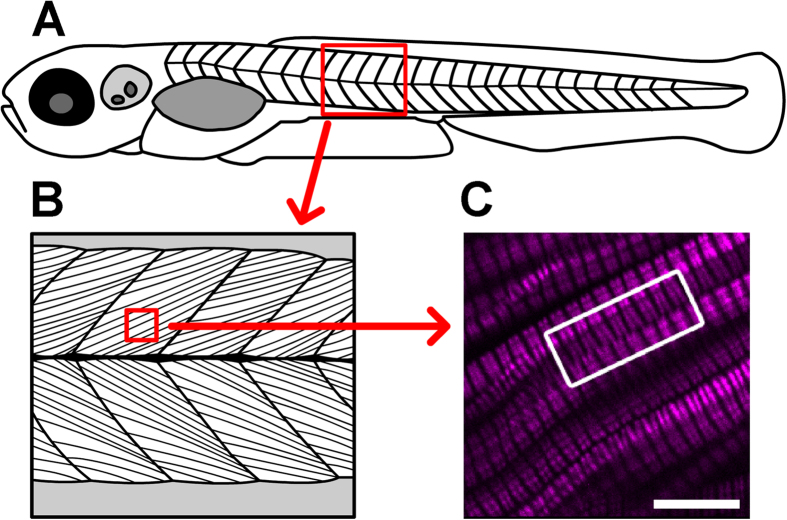
Verniers are regions of distorted endogenous muscle myosin SHG (magenta) that are visible deep within the somite. (**A**) This cartoon depicts a 5 dpf larva (not to scale). The red boxed area highlights a portion of the skeletal muscle compartment, which is illustrated in more detail in (**B**), showing the oriented arrays of muscle cells within the tissue. (**C**) In this image from a 5 dpf, laterally mounted, and fixed WT larva, clear distortions can be appreciated in the SHG signal within muscle cells (curvature toward the edges of the SHG bands in much of the image). The white boxed region draws attention to SHG verniers that appear to span across adjacent muscle cells, suggesting potential physical connections between cells across their membrane boundaries. Scale bar: 10 μm.
