# Supplementary material for: Determination of the source of SHG verniers in zebrafish skeletal muscle
Source: Sci Rep. 2015 Dec 11;5:18119. doi: 10.1038/srep18119 (PMC4676038; doi:10.1038/srep18119)
Supplement: Supplementary Information [file srep18119-s1.pdf]

Supplementary Information

Determination of the source of SHG verniers in zebrafish skeletal muscle

William P. Dempsey[1,*,†], Nathan O. Hodas[2,*], Aaron Ponti[1], Periklis Pantazis[1,‡]

[‡]To whom correspondence should be addressed:

Prof. Dr. Periklis Pantazis

ETH Zurich

Department of Biosystems Science and Engineering (D-BSSE)

Mattenstrasse 26

CH-4058 Basel

Tel. +41 61 387 33 66

Fax +41 61 387 39 93

Email: periklis.pantazis@bsse.ethz.ch

Webpage: www.bsse.ethz.ch/nbi

Supplementary Methods

Calculating Second Harmonic Generation

To study nontrivial geometries, we applied a Green's function approach for calculating SHG[34]:

$$E_{2\omega}(r) = \int G(r,r')P_{NL}(r')\, dr' \quad (1)$$

$$= \int G(r,r') \sum_{jk} \chi_{ijk}^{(2)}(r')E_j(r')E_k(r')dr' \quad (2)$$

where $G(r,r')$ is the Green's function, which corresponds to the wave equation with the specific Green's function determined by boundary conditions, and $\chi_{ijk}^{(2)}$ is the SHG susceptibility tensor. Although this equation is a very general expression, we are concerned with radiation in the far field. Additionally, only light collected by the condenser ever reaches the detector, so - as a result - we are concerned with the angular dependence of the radiation intensity. This simplifies the expression of the Green's function to be approximately independent of near-field boundary conditions[35], and Eq. 2 at a far-field distance, R, becomes[19,36]

$$E_{2\omega}(\Omega, r_0) = [\hat{\boldsymbol{\theta}}, \hat{\boldsymbol{\phi}}] \cdot \frac{\omega^2}{c^2} \frac{e^{ik_{2\omega}R}}{4\pi R} \sum_{jk} \int x^{(2)}(r')E_j(r'-r_0)E_k(r'-r_0)e^{-ik_{2\omega}\mathbf{r}' \cdot \hat{\mathbf{r}}} dr' \quad (3)$$

where the spherical unit vectors are given by

$$\hat{\boldsymbol{\theta}} = cos\theta\ cos\phi \hat{\boldsymbol{x}} + cos\theta\ sin\phi \hat{\boldsymbol{y}} - sin\theta \hat{\boldsymbol{z}} \quad (4a)$$

$$\hat{\boldsymbol{\phi}} = -sin\phi \hat{\boldsymbol{x}} + cos\phi \hat{\boldsymbol{y}} \quad (4b)$$

$$\hat{\boldsymbol{r}} = sin\theta\ cos\phi \hat{\boldsymbol{x}} + sin\theta\ sin\phi \hat{\boldsymbol{y}} + cos\theta \hat{\boldsymbol{z}}, \quad (4c)$$

By convention, we will take the laser's focus to be r_0, which radiates in the z-axis, meaning $\hat{\boldsymbol{k}}_{\boldsymbol{\omega}} = \hat{\boldsymbol{z}}$. We will take the electric field to be polarized in an arbitrary $\hat{\boldsymbol{u}}$ direction and to be a Gaussian mode.

Because the incoming total power is normalized, $E_0 = \sqrt{P/(n_\omega^2 b)}$, where P is the power detected at the back aperture of the objective, we may combine this with the Gaussian mode, and Eq. 3 becomes

$$E_{2\omega}(\Omega, r_0) = [\hat{\boldsymbol{\theta}}, \hat{\boldsymbol{\phi}}] \cdot \frac{\omega^2}{c^2} \frac{e^{ik_{2\omega}R}}{4\pi R} \sum_{jk} \hat{\boldsymbol{x}}_{\boldsymbol{j}} \cdot \hat{\boldsymbol{u}} \hat{\boldsymbol{x}}_{\boldsymbol{k}} \cdot \hat{\boldsymbol{u}} \int x^{(2)}(r') \times$$

$$\frac{P}{n_\omega^2 b} exp\left[-2\frac{(x-x_0)^2+(y-y_0)^2}{\omega_0^2(1+\frac{2i(z-z_0)}{b})}\right] \frac{e^{-i2k_\omega(z-z_0)-ik_{2\omega}r'\cdot\hat{r}}}{(1+2i(z-z_0/b)^2} dr', \quad (5)$$

where $\hat{\boldsymbol{x}}_j \cdot \hat{\boldsymbol{u}}$ is the projection of the polarization vector onto the various Cartesian unit vectors.

Difference between SHG and Fluorescence

To illustrate fundamental differences between two photon excited fluorescence (TPEF) and SHG, we simulated these two processes from idealized theoretical muscles. We constructed the muscle as follows: Given that myofibers are long compared to the focal width of the Gaussian beam propagating in the z-direction, we took them to be effectively infinite in extent in the y-direction. Specifically, myosin radiates in opposite directions from the M-line within each sarcomere. To capture this quality, let $Sq(x, d)$ be a square wave with period 2π, duty cycle $d\%$ and alternating between 1 and -1. Then, assuming $\Delta z < z < \Delta z + w_s$, where $\Delta z = 0.1$ μm is the spacing between sarcomeres and $w_s = 1$ μm is the width of the sarcomere, the spatial density of the SHG susceptibility, $M(x, z)$, is given by

$$M(x, z) = max[Sq(2\pi x/p, d), 0]Sq(2\pi x/p, d/2), \quad (6)$$

where $p = 5$ μm is the length of the sarcomere. For the purpose of illustration, we chose $d = 80\%$. This value specifies the susceptibility of a single myocyte (as in Fig. 2A), and each point is assigned a susceptibility of 1, -1, or 0 for the calculations. For an adjacent, second myofiber, a phase factor of π is added to the square wave periodicity, to represent imperfect alignment between fibers.

We compared SHG signal production to expected signal from TPEF of labeled myosin molecules in the model, since the expected signal from each of these optical processes may differ significantly. To illustrate the signal arising from a fluorescently tagged muscle myosin, we calculated the observed signal that arises when the muscle, represented by $M(x, z)$, is imaged using a two photon microscope.

Investigating the source of SHG verniers

We considered a numeric map of the nonlinear susceptibility of a material (in this case, myosin within zebrafish musculature) — simplified for the purposes of the calculation — given by $M(x, z)$, where $\hat{\boldsymbol{z}}$ is the direction of propagation. To construct a hypothetical muscle, we mapped out myofibers as discrete rectangles of myosin, whose size and shape mirror the arrangement of the thick filaments within sarcomeres. The myosin blocks are arranged in individual columns, each representing a single myofiber, as depicted in Fig. 2B. Because myosin thick filaments project from the M-line, which is at the center of the sarcomere, each half-sarcomere has an oppositely signed susceptibility[18], which is represented by a difference in color on either side of the M-line in Fig. 2B.

The susceptibility tensor for myosin, $x_{ijk}^{(2)}$, has a number of restrictions due to the underlying symmetry of the protein structures (18), leaving only two independent terms, $x_{xxy}^{(2)} = x_{xyx}^{(2)} = x_{zyz}^{(2)} = x_{zzy}^{(2)} = x_{yzz}^{(2)} = x_{yxx}^{(2)}$ and $x_{yyy}^{(2)}$. Experiments have shown that $x_{yyy}^{(2)} < x_{yxx}^{(2)}$, and previous studies have focused on determining different tensor components in SHG signal production[8,37-40].

For the purposes of illustrating SHG in hypothetical muscle, we set up the theoretical model to probe a single tensor component, $x_{yxx}^{(2)}$ (referred to as $x^{(2)}$ for simplicity). As a consequence of these conditions, the possible values of *M(x,z)* represent an effective scalar susceptibility, either 1, 0, or -1 (1). Note that a consideration of symmetry is fundamental to the second-order nonlinear susceptibility, where inversion due to a change of parity is $x^{(2)}(-x) = -x^{(2)}(x)$. If the underlying material possess inversion symmetry, then $x^{(2)} = 0$. Given that the M line contains myofibers extending out in opposite directions, the effective symmetry constraint results in negligible SHG from the M line. To complete the model, hypothetical myofibers were arranged in parallel, and two adjacent muscle cells were placed 180º out of phase with each other.

A paraxial approximation of the wave equation — which has been shown to be an accurate model of the total power produced by an SHG process[19,34,41]— results in the following description of a focused laser propagating in the z-axis using a Gaussian envelope:

$$E_\omega = \hat{u} E_0 \frac{exp\left[-\frac{x^2+y^2}{\omega_0^2(1+i\xi)}\right]}{1+i\xi} e^{ik_\omega z}, \qquad \text{(ref. [7])}$$

where we have adopted the notation of ref. [1], and we define $\xi = \frac{2z}{b}$, where b is the confocal parameter, $b = k\omega_0^2$, and ω_0 is the radius of the beam at the focus.

Given the myosin map detailed above, which we call $\chi(r)M$ (depicted in Fig. 2B), we calculated the two-photon excited fluorescence (TPEF) power by calculating the convolution $\chi M \circ |E|^2$ via Euler integration. Similarly, to calculate the SHG power, we integrated and squared Eq. 5 (see above) using χM. In particular, we used n_{ω} = 1.34, $n_{2\omega}$ = 1.54, NA = 0.8 and λ = 850 nm. n_{ω} and $n_{2\omega}$ were chosen according to analysis in ref. [42].

To estimate the SHG susceptibility tensors in the 3D experimental images, we also developed a segmentation algorithm to select out TPEF signal from fluorescently labeled sarcomeric proteins — in this case, a labeled M-line component in the sarcomere. We estimated the location and orientation of SHG-capable myosin bundles within the sarcomere using this TPEF signal. The estimated 3D arrangement of myosin then determined the resulting estimated SHG susceptibility tensor at each point, $\chi_{est}(r)$. Again, we combined $\chi_{est}(r)$ with Eq. 5 to estimate the SHG power at each point. Because of underlying heterogeneity of zebrafish tissue, important SHG determining factors, including phase-matching (Δk), numerical aperture, and polarizations may be effectively different from experimental design parameters. As a result, we iterated over phase-matching, numerical aperture and polarization angles to find parameters producing the best match to experimentally observed SHG features. The final chosen parameters were NA=0.55, Δk = 0.4, and $\hat{u} = [0.2,0.1,0.2]$.

Zebrafish preparation

Zebrafish husbandry was performed as previously described[31]. Zebrafish embryos were obtained by crossing males and females of the same genetic background. Labeling

of membranes in Fig. S2 was facilitated by zygote stage injections of a membrane-targeted Dendra2 fluorescent protein plasmid, as previously described[43]. F1 PhOTO-N zebrafish[20,21] larvae were used to generate Fig. 3. Briefly, zebrafish were crossed and exposed to 1x1-phenyl 2-thiourea (PTU, Sigma Aldrich; St. Louis, MO) (0.003% W/V) in egg water after 24 hours post fertilization to inhibit the formation of pigment. Fish were kept at 28 ºC in egg water with 1x PTU until imaging (exchanging the PTU solution once every day or two).

Fixed larvae were anesthetized in 0.03% MS-222 (Finquel, Argent Laboratories; Redmond, WA) before being placed in 4% methanol-free formaldehyde (Thermo Scientific; Waltham, MA) on ice for 5 min. The zebrafish larvae continued to fix at room temperature on a nutator (BD Diagnostics; Sparks, MD) for 1 hour after this initial ice incubation before being washed in Ca^{2+}/Mg^{2+}-free 1x PBS 3 times (15 min, each). The fixed animals were then prepared for imaging as described in the following section. Before imaging, live zebrafish were anesthetized using 0.015–0.03% MS-222 in 30x Danieau's solution[32] + 1x PTU.

Zebrafish imaging

Live larval zebrafish were embedded in 1% low melting point agarose (with 1x PTU and 0.015-0.03% MS-222 for living larvae) within 2-well coverslip chambers (Nalge Nunc International; Rochester, NY). Embryos were oriented laterally near the coverslip surface before the agarose reached its full gel strength. Images were obtained using an LD C-Apochromat 40x/1.1 NA water objective (Zeiss) (Fig. S1) or an LD LCI

Plan-Apochromat 25x/0.8 NA Imm Corr DIC multi-immersion objective (Zeiss; Jena, Germany) (Fig. S2).

Fluorescence was captured in the epi-direction using standard filters in the commercial confocal microscope (Zeiss LSM 710 or 780 system), while SHG was captured in the transmitted direction using custom filters (kindly provided by Semrock, Inc.; Rochester, NY) for the selection of the SHG signal peak wavelength. Optical cross section images were obtained using line scanning mode in the 'xz' direction in the case of Fig. 3. For our purposes, a single wide emission filter captured both the fluorescence from the membranes as well as the nuclei in each individual image of PhOTO-N zebrafish larvae. Images were processed in Adobe Photoshop CS3 (Adobe Systems; New York, NY), which included linear contrast and brightness adjustments as well as false color adjustments in ImageJ and Photoshop.

Post-processing of imaging data in Fig. 5

Average masked data was segmented and the boundaries were traced using a custom MATLAB script. Since the coordinates of pixels along the fluorescence boundaries fell on a discrete lattice, the boundary profile was jagged. To prevent artifacts in subsequent calculations, the data was smoothed with a Savitzky-Golay FIR filter[44]. We only considered SHG-capable signal to be present within the sarcomeric banded structure, which is antiparallel to the long axis of each muscle fiber. Thus, we excluded the high-curvature regions at the lateral extremes of the segmented TPEF boundary (corresponding to the short axes of each muscle fiber), ending up with two roughly parallel lines on either side of each TPEF banded signal pattern. Our initial attempt to use both line segments for

the extraction of the local spatial orientation of the muscle structure (see next section) revealed that our simulated SHG signal was rather sensitive to the inaccuracies in the segmentation steps. For each TPEF signal band, we thus removed the lower (i.e., more posterior) segmented line from each pair representing the signal from a single M-line in the analysis. From here, we approximated the remaining anterior lines as the M-lines of each sarcomere, just translated two-dimensionally from the exact center of each sarcomere.

Extracting Normals from post-processed Fig. 5 imaging data

For each pixel along each segmented M-line, two vectors were calculated in three dimensions to define the normal direction (roughly parallel to the long axis of each muscle fiber). The lateral vector was calculated as $\boldsymbol{dx}_i = (\boldsymbol{x}_{z,i+1} - \boldsymbol{x}_{z,i-1})$ and the axial vector was calculated as $\boldsymbol{dz}_i = (\boldsymbol{x}_{z+1,\hat{\imath}} - \boldsymbol{x}_{z-1,\hat{\imath}})$, where z is the index of current plane; $\boldsymbol{x}_i$ is the lateral coordinate $\boldsymbol{x}_i = (x, y, z)$ of the current pixel along the M-line; $\boldsymbol{x}_{z+1,\hat{\imath}}$ and $\boldsymbol{x}_{z-1,\hat{\imath}}$ are the coordinates of the closest positions along the matching M-line in the $z + 1$ and $z - 1$ planes, respectively. The aspect ratio between axial and lateral dimensions matched the ratio of the sampling distances of the fluorescence images (250 and 132 nm, respectively). Since the muscle structures were slightly tilted along the acquisition axis (and thus shifted at different values of z), we corrected for the tilt angle to correctly determine the positions $\boldsymbol{x}_{z-1,\hat{\imath}}$ and $\boldsymbol{x}_{z+1,\hat{\imath}}$ needed to calculate $\boldsymbol{dz}$. As a consequence, all lateral distances between the centers of mass of all M-lines in $z - 1$ and those in z were calculated. To each M-line in $z - 1$, the closest M-line in z was assigned. The global lateral displacement of the tissue was then estimated as the median

of all minimum lateral M-line distances. The same was repeated for planes z and $z + 1$. To find $\boldsymbol{x}_{z+1,\hat{\imath}}$ and $\boldsymbol{x}_{z-1,\hat{\imath}}$ for a given $\boldsymbol{x}_{z,i}$, (i) all boundaries in $z - 1$ and z were corrected for the global displacement, (ii) the closest position to $\boldsymbol{x}_{z,i}$ was found in $z - 1$ and $z + 1$, and (iii) the global displacement was subtracted to restore the original positions to be used to calculate $\boldsymbol{dz}$. The normal vector to the M-line was then calculated as $\boldsymbol{n} = \boldsymbol{dx} \times \boldsymbol{dz}$ and was normalized to unit length. To represent the anti-parallel arrangement of myosin bands on either side of the M-line, we took the calculated normal at the M-line and rotated it by π radians. Finally, to map out the susceptibility from the normals, we extrapolated a vector along the normal for 0.4 microns in both directions to fill a 3D volume.

Supplementary References

34. Kleinman, D. A., Ashkin, A. & Boyd, G. D. Second-Harmonic Generation of Light by Focused Laser Beams. *Phys. Rev.* **145,** 338–379 (1966).
35. Jackson, J. D. *Classical Electrodynamics, 3rd ed.* (John Wiley & Sons, 2007).
36. Mertz, J. & Moreaux, L. Second-harmonic generation by focused excitation of inhomogeneously distributed scatterers. *Optics Communications* **196,** 325–330 (2001).
37. Schürmann, S., Wegner, von, F., Fink, R. H. A., Friedrich, O. & Vogel, M. Second harmonic generation microscopy probes different states of motor protein interaction in myofibrils. *Biophysical Journal* **99,** 1842–1851 (2010).
38. Chu, S.-W. *et al.* Studies of chi(2)/chi(3) tensors in submicron-scaled bio-tissues by polarization harmonics optical microscopy. *Biophysical Journal* **86,** 3914–3922 (2004).
39. Nucciotti, V. *et al.* Probing myosin structural conformation in vivo by second-harmonic generation microscopy. *Proceedings of the National Academy of Sciences* **107,** 7763–7768 (2010).
40. Theodossiou, T. A., Thrasivoulou, C., Ekwobi, C. & Becker, D. L. Second harmonic generation confocal microscopy of collagen type I from rat tendon cryosections. *Biophysical Journal* **91,** 4665–4677 (2006).
41. Kleinman, D. A. & Miller, R. C. Dependence of Second-Harmonic Generation on the Position of the Focus. *Phys. Rev.* **148,** 302–312 (1966).
42. Hodas, N. O. Nonlinear dynamics of nanoscale systems. (2011).
43. Caneparo, L., Pantazis, P., Dempsey, W. & Fraser, S. E. Intercellular bridges in vertebrate gastrulation. *PLoS ONE* **6,** e20230 (2011).
44. Savitzky, A. & Golay, M. J. E. Smoothing and Differentiation of Data by

Simplified Least Squares Procedures. *Anal. Chem.* **36,** 1627–1639 (1964).

Supplementary Figures

Supplementary Fig. 1: Verniers appear in the SHG channel when myosin bands are staggered in adjacent myofibers. (A) In this simplified two-dimensional cartoon, two myofibers are depicted next to one another (sarcolemma plasma membranes are labeled as dashed lines surrounding the actin/myosin bundles), and their individual myosin (black, thick filaments) and actin (gray, within thin filaments) sarcomere bands have offset sarcomere banding patterns with respect to each other. Note that the Z-lines of each sarcomere in the myofiber are depicted as black lines in the actin bands, while the M-lines are depicted as gaps at the center of the myosin bands. **(B)** In the SHG channel, theory predicts that the myosin signal (red) may form verniers and could even seem to link the adjacent myofibers in some regions. **(C)** In contrast, when the myosin is fluorescently tagged, TPEF signal emanating from labeled myosin (green) would remain basically rectangular in shape, with no gaps in fluorescence at the M-lines. Note that in panels B and C, the actin bands are assumed to be unlabeled, which is why they are not shown in the images. Fluorescence from labeled actin bands would look similar to panel C, displaying no vernier patterns. This cartoon is not to scale, and 2P illumination is assumed to be in the direction from the top to the bottom of each panel. This figure is for illustrative purposes only and does not represent actual results from the theoretical simulations, as depicted in Fig. 2 in the main text.

Supplementary Fig. 2: Myosin-derived SHG vernier patterns vary depending on the orientation of adjacent myofibrils. In the same hypothetical muscle tissue modeled in Fig. 2 in the main text, the positions of the two rectangular blocks of myosin (see Fig.

2B) were varied to produce staggered orientations with differing phase. The white dashed line in each panel represents the muscle cell boundary. **(A)** When the phase difference between myofibers is zero (i.e. the myosin blocks are completely aligned), no vernier patterns cross the cell boundary. However, at phase differences of **(B)** $\pi/4$, **(C)** $\pi/2$ and **(D)** $3\pi/4$, illusory vernier patterns are visible, linking the adjacent myofibril blocks.

Supplementary Fig. 3: Endogenous SHG imaging facilitates monitoring of muscle organization in vivo with subcellular resolution. These panels display a single optical section image of the trunk within a laterally mounted, live, and membrane-targeted fluorescent protein labeled (see Supplementary Methods) zebrafish embryo at 2 dpf. **(A)** A merged image of SHG signal (white) and fluorescence from the labeled membrane (green), showing the intracellular striated SHG pattern arising from endogenous sarcomeric myosin bundles within the myofibers. The cartoon inset depicts an embryonic zebrafish during segmentation, and the highlighted region indicates the general vicinity of imaging. **(B)** In the SHG signal channel alone, the striated SHG pattern can be appreciated more clearly. The somite boundaries, the vertical myosepta, are clearly visible in the image (dark boundaries separating the longitudinal muscle fibers). Certain pigments are visible as broadband signal overlapping with the SHG channel (arrow) at this stage of development. Scale bar: 30 μm.

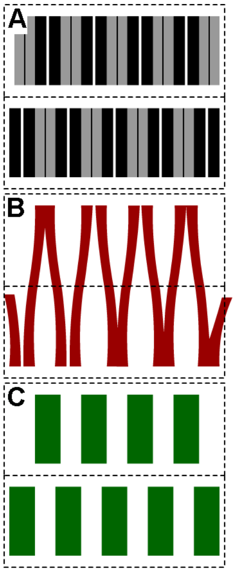
A
B
C

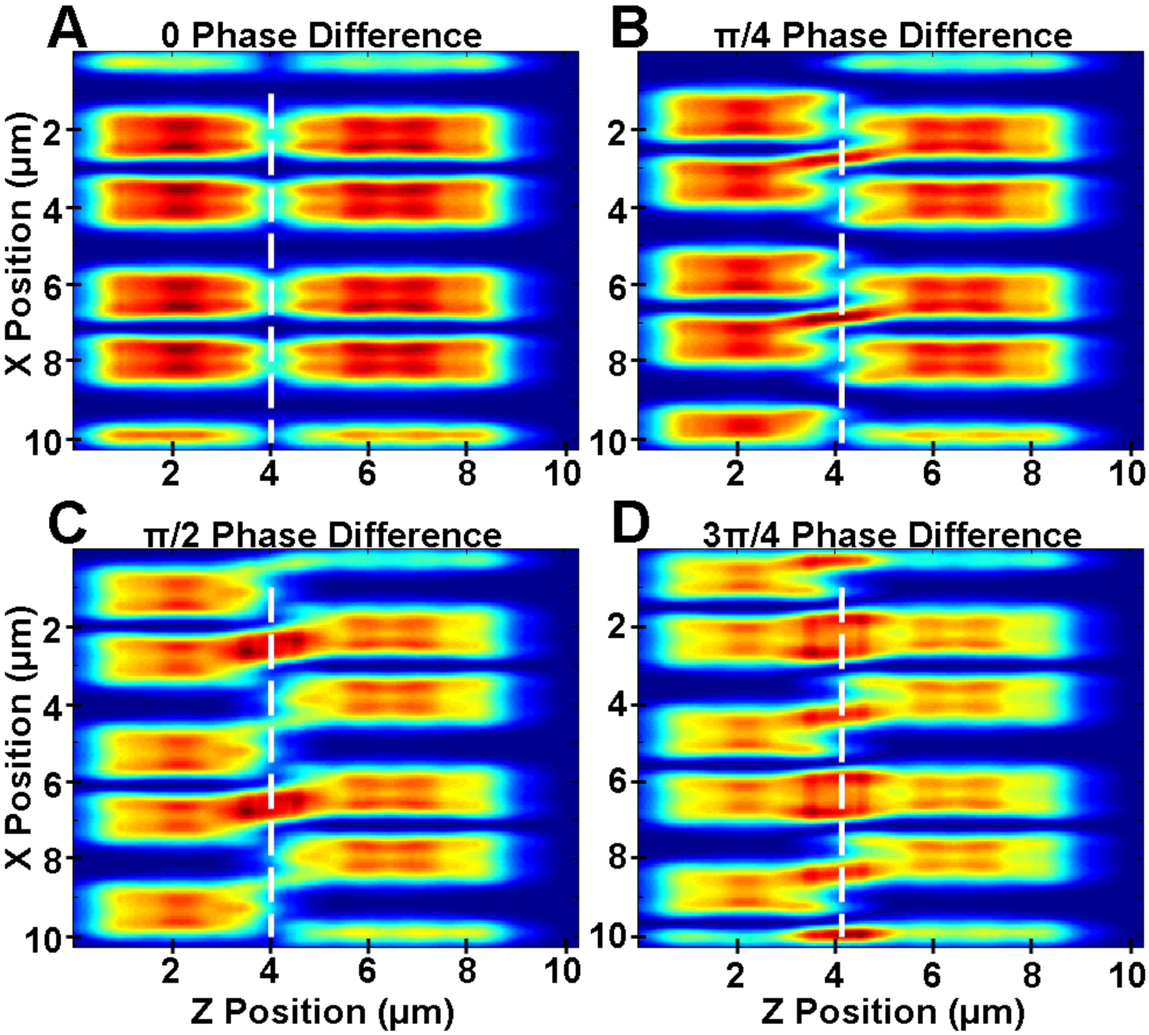

A
0 Phase Difference
B
π/4 Phase Difference
C
π/2 Phase Difference
D
3π/4 Phase Difference
X Position (μm)
Z Position (μm)
2
4
6
8
10

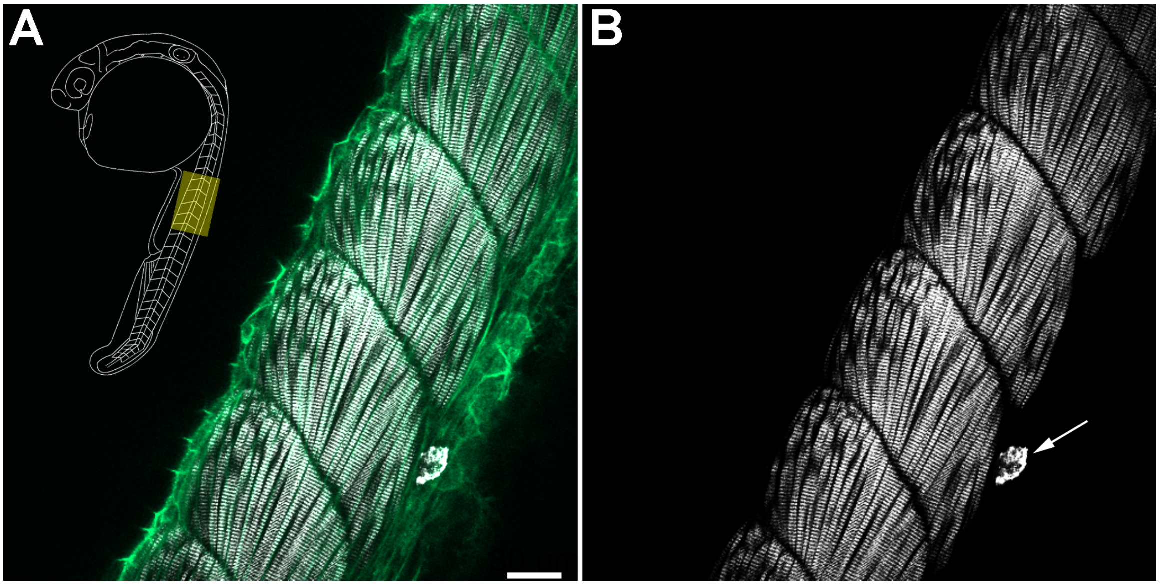
A
B
